# Supplementary material for: COPS5 is Essential for Sertoli Cell Function and Male Fertility in Mice
Source: bioRxiv. 2025 Dec 22:2025.12.19.695357. Preprint. [Version 1] doi: 10.64898/2025.12.19.695357 (PMC12776080; doi:10.64898/2025.12.19.695357)
Supplement: Supplement 1 [file NIHPP2025.12.19.695357v1-supplement-1.pdf]

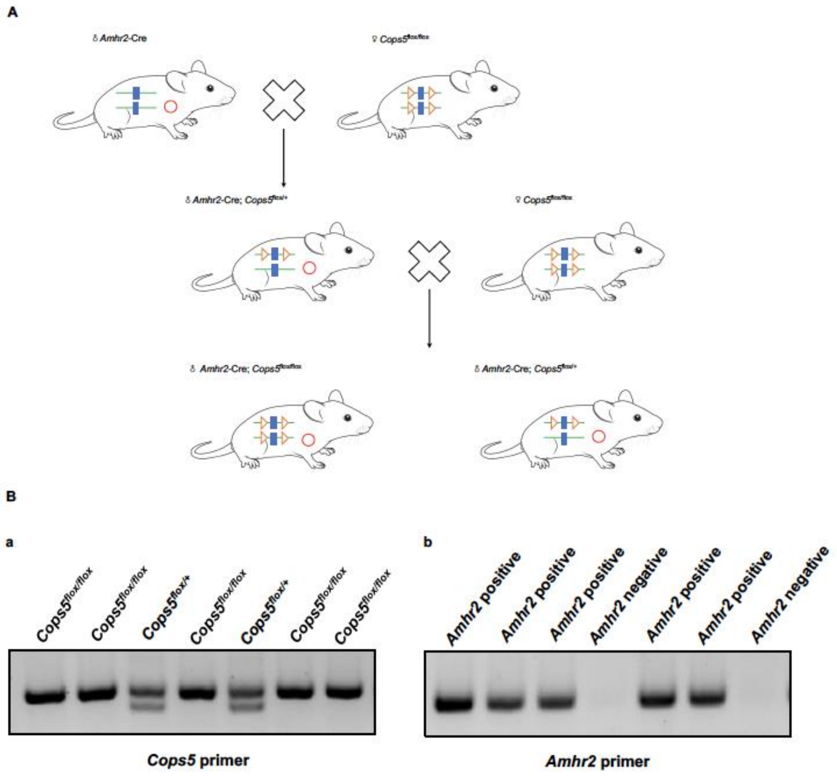
A
♂ Amhr2-Cre
♀ Cops5flox/flox
♂ Amhr2-Cre; Cops5flox/+
♀ Cops5flox/flox
♂ Amhr2-Cre; Cops5flox/flox
♂ Amhr2-Cre; Cops5flox/+
B
a
Cops5flox/flox
Cops5flox/flox
Cops5flox/+
Cops5flox/flox
Cops5flox/+
Cops5flox/flox
Cops5flox/flox
Cops5 primer
b
Amhr2 positive
Amhr2 positive
Amhr2 positive
Amhr2 negative
Amhr2 positive
Amhr2 positive
Amhr2 negative
Amhr2 primer

bioRxiv preprint doi: https://doi.org/10.64898/2025.12.19.695357; this version posted December 22, 2025. The copyright holder for this preprint (which was not certified by peer review) is the author/funder, who has granted bioRxiv a license to display the preprint in perpetuity. It is made available under aCC-BY 4.0 International license.

Supplemental Figure 1. Generation of Sertoli cell-specific *Cops5 KO* mice.

A. Breeding strategy to generate *Cops5* cKO mice;

B. Representative PCR result for mouse genotyping.

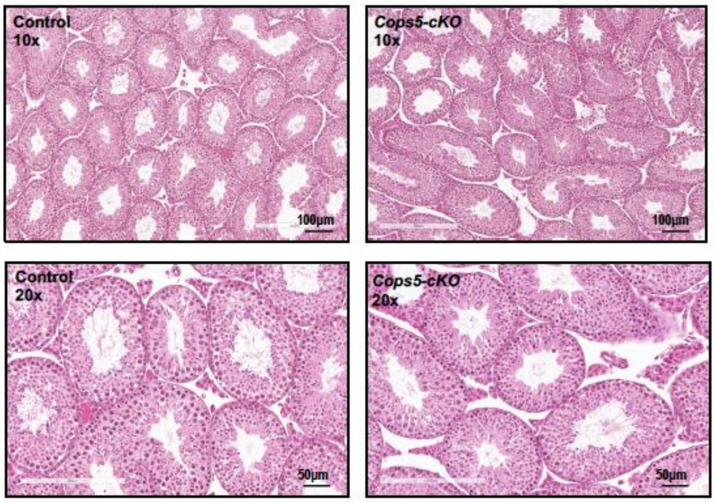

Supplemental Figure 2. Testis histology of 6-week-pld control and *Cops5* cKO mice.

Representative images of H&E staining of testis from 6-week-old control and *Cops5* cKO mice. No difference was observed between the two genotypes.

bioRxiv preprint doi: https://doi.org/10.64898/2025.12.19.695357; this version posted December 22, 2025. The copyright holder for this preprint (which was not certified by peer review) is the author/funder, who has granted bioRxiv a license to display the preprint in perpetuity. It is made available under aCC-BY 4.0 International license.

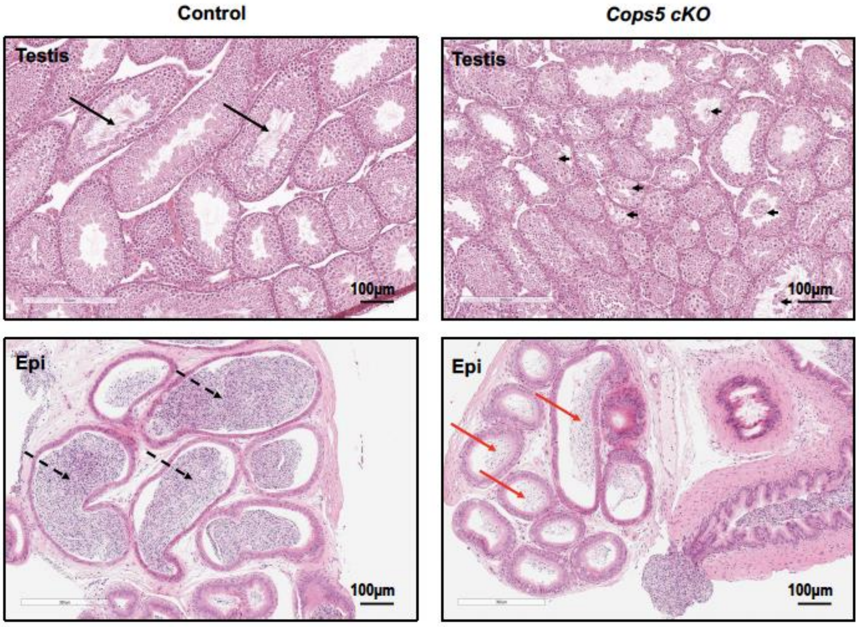

Supplemental Figure 3. Testis histology of 3-month-old control and *Cops5* cKO mice at low magnification.

The black arrows point to the sperm in the seminiferous tubule lumen in the control mice. The dashed arrows point to the sperm in the cauda epididymis lumen of the control mice; the arrow heads point to the degenerated germ cells in the seminiferous tubule lumen in the *Cops5* cKO mice; the red arrows point to the few sperm in the cauda epididymis lumen of *Cops5* cKO *mice.*
